# Extralobar pulmonary sequestration with absence of pericardium and atrial septal defect in a woman

**DOI:** 10.1186/s13019-019-0932-9

**Published:** 2019-06-20

**Authors:** Zhenhuan Tian, Yuncan Zhou, Hongsheng Liu

**Affiliations:** 1 Department of Thoracic surgery, Peking Union Medical College Hospital, Peking Union Medical College, Dongcheng District, Beijing, 100730 China; 2 Peking Union Medical College, Beijing, China

**Keywords:** Extralobar sequestration, Congenital absence of the pericardium, Atrial septal defect, Uniportal video-assisted thoracoscopic surgery

## Abstract

**Background:**

Extralobar sequestration is a rare congenital malformation of lung tissue, which can be combined with other foregut and cardiac abnormalities. Our case is the first to report extralobar sequestration, absence of pericardium and atrial septal defect in the same patient.

**Case presentation:**

A 22-year-old female with atrial septal defect came for her recent atypical symptom of intermittent palpitation and shortness of breath. Her computed tomography showed a cystic mass located in left superior anterior mediastinum near the pulmonary trunk. With specious of cystic teratoma prior to video-assisted thoracoscopic surgery, she finally was diagnosed as extralobar sequestration, while incidentally found with congenital absence of pericardium during surgery.

**Conclusions:**

Extralobar sequestration, absence of pericardium and atrial septal defect can occur in the same patient. The preoperative diagnostic rate of extralobar sequestration and asymptomatic absence of pericardium remains low attributed to atypical imaging features. A cardiac magnetic resonance imaging is highly recommended if necessary. Regular follow-up is essential to asymptomatic absence of pericardium and atrial septal defect patients. To patients with extralobar sequestration, an operation could be performed.

## Background

Extralobar sequestration (ELS) is a rare congenital malformation of lung tissue with the prevalence from 0.16 to 6.4%, men to women is about 3:1 [1]. It is a non-functional lung mass with no normal connection with tracheobronchial tree. Here we report a case of ELS whose preoperative diagnosis was cystic teratoma. It also has concurrent congenital absence of pericardium (CAP) and atrial septal defect (ASD).

## Case presentation

**Fig. 1:**
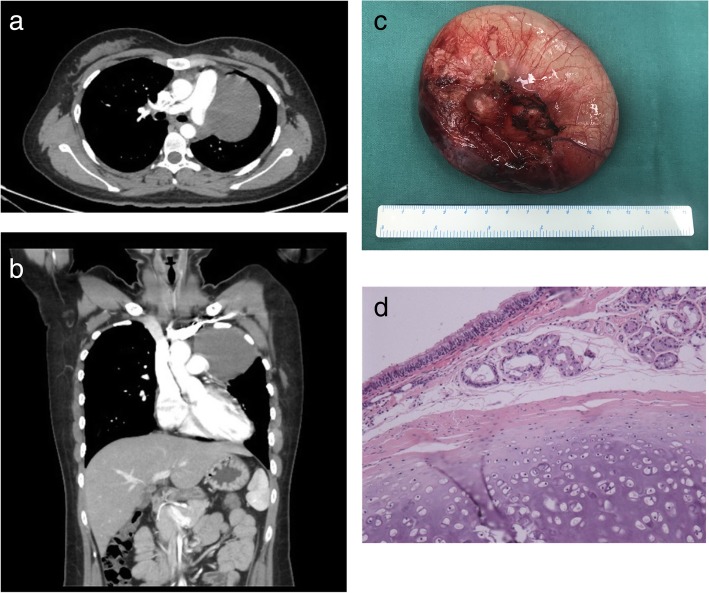
Enhanced CT scan, gross morphology and microscopic appearance of the lesion. (**a**. cross-section view in CT scan). (**b**. coronal view in CT scan). (**c**. gross morphology of the large, well-defined, cystic lesion). (**d**. microscopic appearance showing cystic dilated bronchus and lung tissue (haematoxylin–eosin stain, 100 × magnification))

**Fig. 2:**
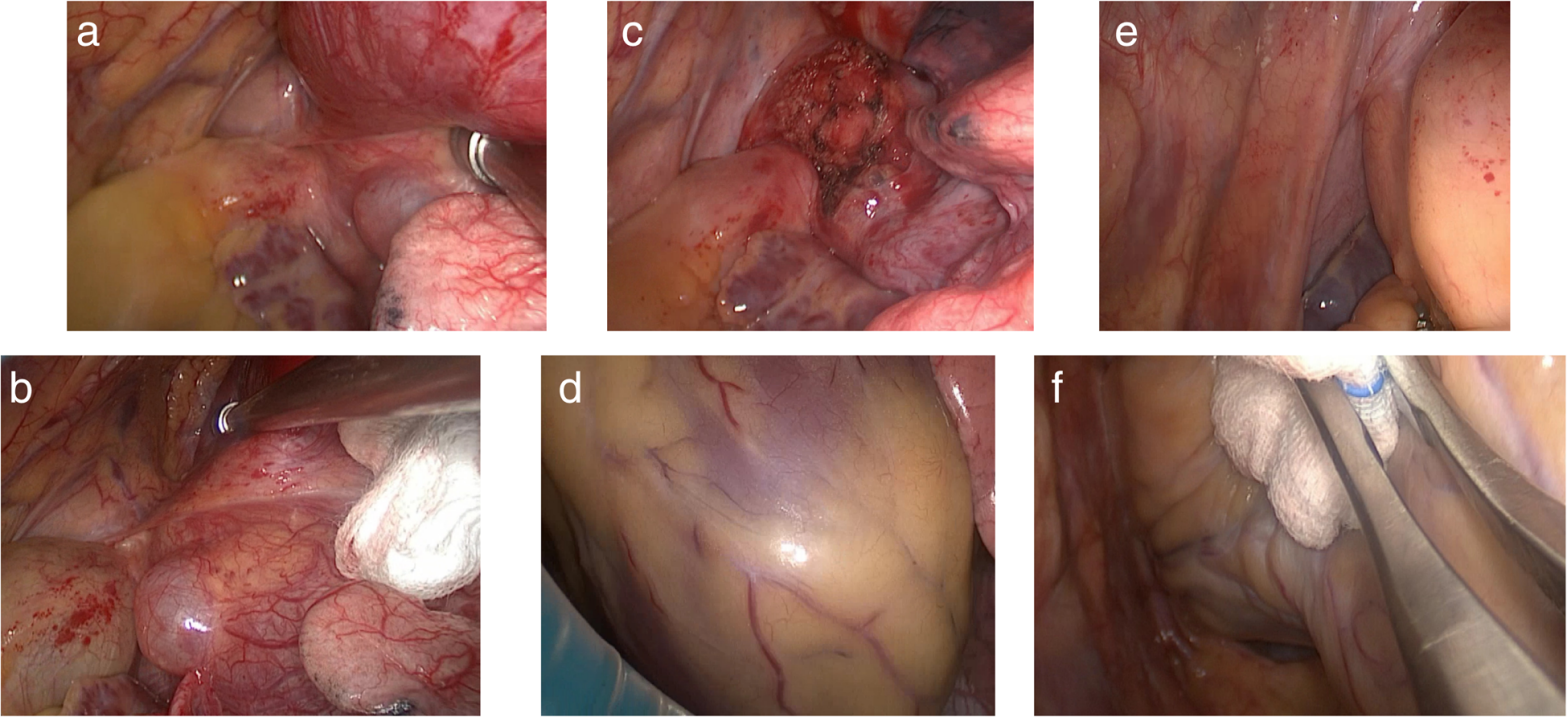
Surgery images. (**a**, **b**. the mass, arcus aortae, left atrial appendage). (**c**. postoperative surgical site). (**d**. heart). (**e**. left phrenic nerve, right atrial appendage). (**f**. right thoracic cavity)

A 22-year-old woman came to our department with complaints of intermittent palpitation and shortness of breath for 6 months. Physical examination was unremarkable. Echocardiography revealed that there was an ASD with the diameter of 10 mm. Enhanced computed tomography (CT) demonstrated a huge well-defined and homogeneous cystic mass, in left superior anterior mediastinum and in close proximity to pulmonary trunk (Fig. 1a and b), no significant artery feeding were found. Cancer antigen 125 was higher than normal. The uniportal video-assisted thoracoscopic surgerywas performed with the preoperative diagnosis of cystic teratoma. The first striking intraoperative finding was the completely CAP. A large well-defined cystic lesion (9 cm × 9 cm × 10 cm) was visualized, which bulged through the aortopulmonary window (Fig. 2). We performed a mini-incision to cystic lesion, from which large amounts of white viscous liquid was sucked. Finally the basal segment of mass was separated carefully from mediastinal pleura (Fig. 1c). Besides, we did not do surgical intervention to CAP and ASD. Histopathology revealed that grossly, the lesion had a smooth inner wall with gray gelatinous material. And cystic dilated bronchus, lung tissue and its own pleura could be seen under microscope (Fig. 1d). The final diagnosis of this patient was ELS, not cystic teratoma. We reassessed the enhanced CT, but we still could not found a significant feeding artery for ELS.The patient recovered well and in excellent condition during follow-up,with no intermittent palpitation and shortness of breath.

## Discussion and conclusions

ELS usually occurs between left lower lobe and diaphragm [2]. It mostly manifests in infancy stage and approximately 60% of ELS is associated with other congenital abnormalities, such as foregut malformations and cardiac anomalies [3]. In our case, the patient is a young adult woman with a mass located in superior anterior mediastinum, which is particularly unusual in gender, onset age and pathogenic site. Moreover, she also has concurrent CAP and ASD. Only three cases about ELS accompanied by CAP have been reported up to now [4–6]. Our case is the first to report ELS, CAP and ASD at the same time. The association between pathogenesis of ELS, CAP and ASD has not been clarified yet.Clinical manifestation of ELS include non-specific symptoms due to secondary pulmonary infection, mass effect and congenital anomalies-related symptoms. There are also a few asymptomatic patients having been diagnosed incidentally [2]. Our patient had no signs of infection and her symptoms were most probably attributable to mass effect. According to the rarity of ELS, higher prevalence of cystic teratoma in young women, and nonspecific results of her enhanced CT, we regarded cystic teratoma as the first preoperative diagnosis. CAP can be ignored preoperatively even if we have done enhanced CT. The thin fat layer in that position makes it difficult to discover CAP from enhanced CT. But enhanced CT can actually offer some significant evidence, such as levorotation of heart and interposition of lung tissue in regions of absent pericardium, including anterior space between aorta and pulmonary artery. With those signs, cardiac magnetic resonance imaging is a recommended choice, regarded as gold standard [7]. Regretfully, there is not any significant signs either in this patient.

Surgical resection remains the standard treatment for ELS. To symptomatic patients, such as infection or hemoptysis, confine operation is recommended. To asymptomatic patients, selective surgery can be performed under regularly monitoring. Of uncertain cases, surgery is also a proper way to identify uncertain cases [1, 2, 8]. Video-assisted thoracoscopic surgery is as effective and safe as thoracotomy for ELS [1, 8]. For our patient, we only performed a video-assisted thoracoscopic surgery mediastinal cystectomy and did not interfere with CAP and ASD.In conclusion, ELS may be combined with CAP and ASD at the same time. Its preoperative diagnostic rate remains quite low due to nonspecific imaging manifestations. Asymptomatic CAP is also difficult to be discovered. A cardiac magnetic resonance imaging is highly recommended if there are indirect signs. An operation should be performed when diagnose is certain. The most common treatment is regular observation to asymptomatic CAP and ASD. For patients with the complete bilateral or complete left-sided absence of the pericardium, no treatment is indicated [9]. According to the guideline of European Society of Cardiology, We suggested the Interventional therapy for the patient after operation. But the patient refused for her own consideration, we will continue to track the patient’s condition in our clinic.

## Data Availability

The datasets used and/or analysed during the current study are available from the corresponding author on reasonable request.

## Abbreviations

ASD: Atrial septal defect
CAP: Congenital absence of pericardium
CT: Computed tomography
ELS: Extralobar sequestration
